# Four analysis moments for fuzzy cognitive mapping in participatory research

**DOI:** 10.1080/16549716.2024.2430024

**Published:** 2024-12-02

**Authors:** Iván Sarmiento, Anna Dion, Mateja Šajna, Neil Andersson

**Affiliations:** a CIET-PRAM, Faculty of Medicine and Health Sciences, Department of Family Medicine, McGill University, Montreal, QC, Canada; b Grupo de Estudios en Sistemas Tradicionales de Salud, Escuela de Medicina y Ciencias de la Salud, Universidad del Rosario, Bogotá, Colombia; c Department of Mathematics and Statistics, University of Ottawa, Ottawa, ON, Canada; d Centro de Investigación de Enfermedades Tropicales, Universidad Autónoma de Guerrero, Acapulco, México

**Keywords:** Participatory modelling, participatory research, community engagement, contextualization of evidence, family medicine, global health

## Abstract

Fuzzy cognitive mapping (FCM) is a practical tool in participatory research. Its main use is clarifying causal understandings from several knowledge sources. It provides a shared substrate or language for sharing views of causality. This makes it easier for different interest groups to agree what to do next. Each map is a collection of causal relationships with three elements: factors (cause and outcome), arrows linking factors, and weights indicating the perceived influence of each cause on its outcome. Stakeholder maps are soft models of how they see causes of an outcome, such as access to services or systemic racism. Based on a standardized FCM protocol, we present four moments in FCM analysis. (1) Agree shared meaning across maps. (2) Calculate the maximum influence of perceived causes. (3) Simplify the maps for communication. (4) Identify priorities for action. We provide explanations of the four moments in FCM analysis, with examples from five countries. FCM offers a practical means to guide health action. It incorporates local perspectives with transparent and traceable procedures.

## Paper Context

- **Main findings**: We present Fuzzy Cognitive Mapping as a shared platform for analyzing diverse knowledge sources and amplifying the voices of stakeholders in understanding, discussing, and acting on health issues.
- **Added knowledge**: This manuscript addresses many of the questions we frequently receive from researchers who want to conduct participatory research globally.
- **Global health impact for policy and action**: Fuzzy Cognitive Mapping is instrumental in the context of an increasing acceptance that evidence-based medicine, health management, and planning must leverage different types of evidence – some formal and scientific, others local with differences between stakeholders.

## Background

Fuzzy cognitive mapping (FCM) can clarify the understanding of how causes contribute and interact to influence an outcome [1–3]. Applications include environmental science [4], decision-making [5], engineering [6], economics [7,8], organizational behaviour, information technology and, more recently, health care [9,10]. FCM has uses in machine learning, and it offers a computational framework [11,12] for predictive modelling [13–15]. It is also a powerful tool to include voices and knowledge of stakeholders in decision-making, the focus of this paper.

Participatory research uses FCM as soft models of stakeholder causal knowledge [1,16]. From an individual or group perspective, stakeholders map their view of causes of a particular outcome. Views of causality differ from person to person and group to group, and each view is partial and changing over time [17]. FCM provides a shared language for juxtaposing these different views. It allows comparisons and sometimes combination [18,19]. In our practice, FCM focuses on inclusion of under-represented stakeholder knowledge in shared decision-making [20,21]. The output is not a regularity- or variance-based vision of cauzation [18]. Independent of researcher paradigms, FCM portrays knowledges of causes [17] in terms the participants use and understand.

**Figure 1.**
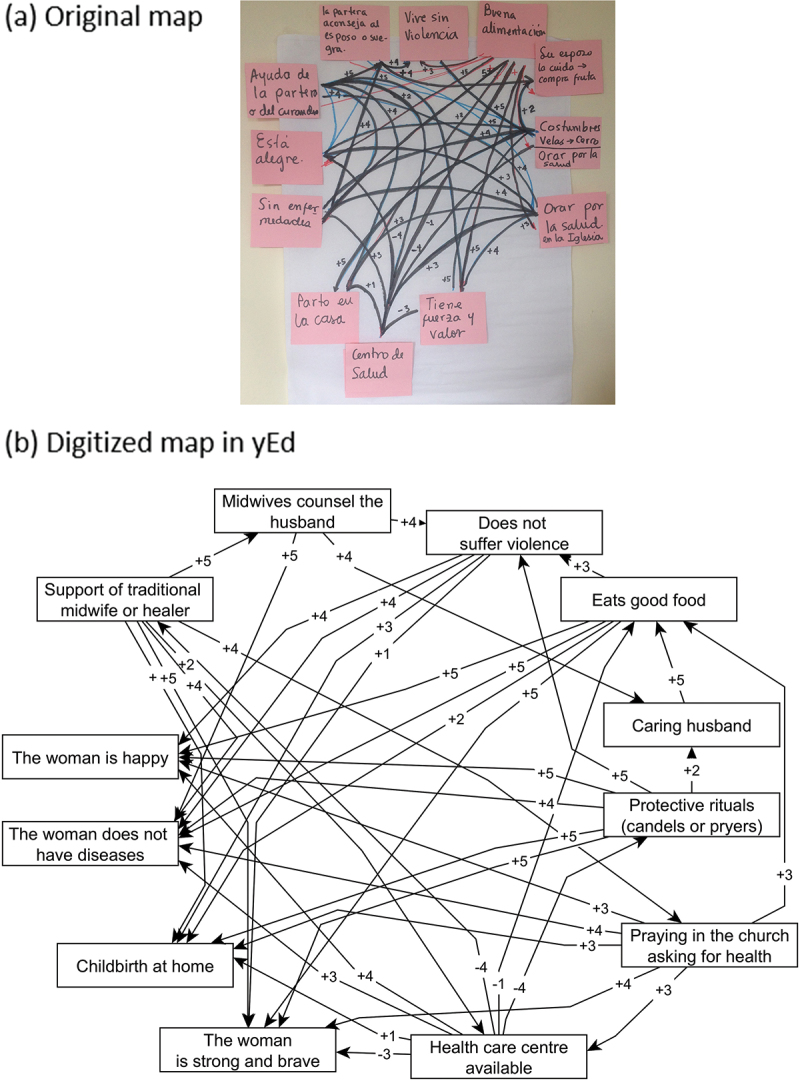
Fuzzy cognitive map of factors contributing to better maternal health according to a group of traditional midwives in Guerrero, Mexico.

Legend. Indigenous traditional midwives in Guerrero, Mexico, drew a fuzzy cognitive map in a four-hour session with the support of a facilitator and two Indigenous intercultural brokers. The map presents protective factors for maternal health according to participants. Traditional midwives disaggregated maternal health into four outcomes: the woman is happy, the woman is strong and brave, the woman can give birth at home, and the woman does not get sick. a) The original map with labels in Spanish, and b) a digitized diagram with labels translated into English. The map has 12 factors (nodes) and 38 relationships (arrows). Appendix 1 includes the tabular representation of the maps as an adjacency matrix and an edge list.

Creating fuzzy cognitive maps is straightforward but requires robust protocols for reproducibility across different settings [16]. Maps have three elements [2,3]. Factors or nodes represent causes and outcomes. Arrows represent relationships or causal links between nodes. Adding *fuzzy* to cognitive mapping, weights reflecting relative strength of influences between nodes. The influence between each cause on an outcome can be direct or indirect through intermediaries [18]. The maps can also reflect cyclic relationships with loops or two-way interactions. Figure 1 shows a fuzzy cognitive map built by traditional midwives in Guerrero, Mexico, to depict how they see contributing factors in healthy maternity in their communities.

Our four analysis moments draw on three graduate courses on advanced methods for participatory research [22], over 200 hours of short courses for international researchers, and participatory research experience in 25 settings [16]. This paper describes the four moments illustrating, with examples, how each builds on earlier moments.

Our work with birth companions and family physicians in Montreal, Canada, contextualised published literature on unmet care needs of recent immigrant women [23]. In the Mexican state of Guerrero, we explored views of *Nancue ñomndaa* and *Me’phaa* traditional midwives on what promotes maternal health [24]. In Nigeria, FCM stimulated discussion about causes of short childbirth interval in Hausa communities [25]. Two examples from Botswana used FCM to map perceived causes of suicide among young men and violence against women [26–28]. We also provide references to other FCM applications that can inform decisions in FCM analysis.

## Methods

There are FCM protocols for different contexts [1,3,21,29]. The Andersson and Silver protocol describes ten steps for creating and analysing maps in participatory research [1]. In this paper, we describe what happens after map creation, to make sense of and to share knowledge on health concerns and how to address them [30]. Analysis of the maps completes the participatory FCM protocol, which includes discussing and interpreting results with stakeholders [25] or contextualizing literature reviews [23].

Fuzzy cognitive maps have different formats of value in analysis. Figure 1 presents the graphic format generated by *Nancue ñom ndaa* midwives in Guerrero, showing the complexity of their knowledge. This is useful for clarifying knowledge and when discussing the maps with a stakeholder group. Appendix 1 presents two additional formats. The *edge list* is a collection of all links on the map, presented as rows in a three-column table. The first column is the origin (where the arrow starts), the second column is the landing (to which the arrow points), and the third column contains the weight of the link between the nodes. Another digital format for FCM data is the *adjacency matrix*, akin to those used in printed road maps: a square table where each row and column corresponds to one node in the map. The value of the cell at the intersection of a row (origin node) and column (landing node) shows the weight of the link between the two [3]. The edge list and adjacency matrix are the input formats for computations with the weighted links in the maps. These tables also help direct data collection. During mapping sessions with stakeholders, following our protocol, we find it useful to record weights from one to five. Not reporting a relationship between two nodes on the map means a weight of zero. For computation, we use probability architecture and scale all weights to the range 0 to 1.

We present four moments in FCM analysis: (1) Agree shared meaning of concepts across maps, (2) Calculate the maximum influence of perceived causes, (3) Simplify maps for communication, and (4) Identify priorities for action. Our usual FCM analysis starts with standardization of node labels between maps and then uses transitive closure before more complex analyses to address the interdependence between factors within maps. Pattern correspondence tables, fuzzy and probabilistic transitive closure combine with social network analysis to make sense of fuzzy cognitive maps from different sources. We propose this as a reproducible and transparent approach to FCM analysis in participatory research using tools that are freely available on http://ciet.org/fcm. This English and Spanish website includes an extensive collection of tools for FCM. Appendix 2 describes other relevant analysis approaches.

### Moment 1: agree shared meanings of concepts across maps

The opening challenge in FCM analysis is understanding the meaning of concepts in stakeholder generated maps. Stakeholders might use different languages or refer to the same factor using different terms. *Standardization* involves identifying all factors sharing the same meaning and renaming them with a standard label or indicative code. This essential step translates content for easy contrast across maps, before digitization. *Iterative standardization* during data collection helps to distinguish overlapping concepts. In practical terms, a debriefing meeting after each mapping session reviews the maps and discussion notes with facilitators. It generates a list of labels facilitators can use in the next mapping session. This small step improves data quality and saves time later during the analysis. Andersson’s protocol emphasises participant involvement in this process [1], as stakeholders are the authoritative sources on meaning of their own words. In Nigeria and Botswana, where logistics limited participant involvement, we relied on detailed notes from the mapping and facilitators who had led the discussions to clarify participant contributions [25–27]. A clear list of decisions and assumptions during the reconciliation of factor meanings is essential for transparency.

Pattern matching in computer science refers to checking a given sequence of tokens, in this case, factors from the maps, for similarities and differences [31]. In FCM analysis in participatory research, pattern *correspondence* allows for visual alignment of different factors across maps. Table 1 juxtaposes maps from two groups of traditional midwives in Guerrero, Mexico [24]. Similar factors align on the same row; blank cells mean the respective stakeholder did not mention the factor.

**Table 1.** Protective factors for maternal health identified midwives in Guerrero, Mexico, and their corresponding categories.

Standard factor name or code	Protective factors enumerated in Community 1	Protective factors enumerated in Community 2	Category/theme
F1	The woman is happy	The woman is happy, beautiful, good worker, not lazy, does not get ‘coraje’. Also, she has a healthy husband	*The woman has a safe birth and healthy maternity*
F2	The woman is strong and brave		
F3	The woman is able to give birth at home	A good childbirth and delivery: healthy pains, less blood loss, fast healing	
F4	The woman does not get sick	Healthy postpartum: healthy baby/the woman is willing to eat after childbirth	
F5	Support of a midwife or traditional healer	The woman receives care from the traditional midwife (and she takes care of the position of the baby)	*The woman has support of a traditional midwife or healer*
F6		Traditional midwives in the community	
F7	A midwife counsels the husband		
F8	Healthcare centres available	Hospital available	*A healthcare centre or hospital is available*
F9	The woman follows protective rituals (lighting candles or indigenous prayers)	The woman follows protective rituals associated with traditional medicine	*The woman follows protective rituals*
F10	Praying in the church (Christian or Catholic) asking for health		
F11		The woman takes care of herself	*The woman follows self-care practices*
F12	The woman does not suffer violence		*The woman does not suffer violence*
F13		The woman lives without worries	*The woman lives without worries*
F14		The woman is well treated by the husband	*The woman has a caring, working, and loving husband*
F15	The woman has a caring and loving husband	The woman has a caring and working husband	
F16		The husband talks to the baby in the womb	
F17		Good communication with husband	*The woman has good communication with husband*
F18		The woman discusses (talks) with husband about pregnancy and delivery	
F19		The woman does not get sick	*The woman has a good health condition (before pregnancy)*
F20		The woman heals from her diseases	
F21		Economic stability	*The woman has economic stability*
F22	The woman eats good (enough) food	The woman eats good (enough) food	*The woman is well nourished*

Legend. Reproduced without change under the authorization of the authors of [24].

At its simplest, pattern correspondence involves ordering rows in the table, clustering factors by stakeholder A, stakeholder B, shared by A and B. Pattern correspondence tables can key off and inform dialogue about similarities and differences without further computation or mathematical algorithms. A first hint of consensus between stakeholders is the proportion of maps identifying a particular factor as a cause of the outcome of interest [32]. Comparing across maps demonstrates how different stakeholders identify different perceived causal factors. This raises useful questions about quite what each factor means, and its importance. Shared views of causes and outcomes provide an easy opening for dialogue about what needs to change. Non-shared views can also be informative about what needs to change. In Nigeria, most women’s groups recognized coerced sex and lack of male involvement in childbirth as important factors in childbirth intervals, yet few groups of men mentioned these factors in their maps [25].

Pattern correspondence works well with a limited number of concepts and not too many maps. Its utility decreases when there are many factors and many stakeholder groups providing maps. A first step is to combine maps from similar stakeholders – all older men, or all younger women. It is also possible to compare maps after making fewer but broader categories of causes (see Moment 3). Additionally, pattern correspondence provides more information after we calculate the maximum strength of influences across knowledge sources.

### Moment 2: calculate the maximum influence of each factor

Stakeholder maps collate a series of direct relationships (node-arrow-node). This makes it difficult to disentangle influences across the whole map. A map might have many different paths for factors influencing other factors. Our second analysis moment aims to understand how each cause influences all other causes, and which has the maximum influence.

*Transitive closure* is computation to contextualize each weighted relationship, accounting for all other nodes through all possible pathways or succession of factors [22]. By linking each factor with all other factors in the map, transitive closure converts a collection of individual associations into a knowledge network. It identifies direct and indirect relationships between each cause-outcome pair. Some causes might not have much influence on their own, but they can form part of a ‘walk’ or chain of relationships that change the overall influence on the outcome [2].

We use two transitive closure procedures: probabilistic and fuzzy. Both follow the same principle, calculating maximum influences through direct and indirect paths, but rely on different assumptions on how to do so [33]. These differences affect how we interpret the findings. We use probabilistic transitive closure in maps made of a fixed number of predetermined factors, since probability calculations depend on the length of the path. In probabilistic transitive closure, the resulting weight of a walk (or sequence of related factors) is the product of the weights of arrows in that walk. An example is our FCM analysis of shifts in the CASCADA behaviour change sequence after an intervention. The CASCADA behaviour change model describes a seven-step results chain between conscious knowledge (the first C of the acronym) and action (the last A of the acronym). Two randomized controlled trials on cultural safety training in medical education in Colombia used FCM with probabilistic transitive closure to compare the differences in the influence of these intermediate outcomes between intervention and control groups [34,35].

Fuzzy transitive closure is a better fit when stakeholders define the scope of factors to include in the maps – some map authors might mention ten factors while others mention 5 or 50. Probability models for weighting these maps are difficult. The inter-dependence of factors in these maps relies on the level of detail participants provide. There are differences between stakeholder views. But there are also random determinants, like time available for mapping, number of people in the group or the weather, which affect how many factors map authors name. Fuzzy transitive closure calculates the weight of a walk (or sequence of related factors) as the minimum weight among all the arrows participating in the walk [2]. In complex soft models with unwieldy amounts of information on what causes a single outcome, transitive closure identifies the most influential causal pathways [1].

**Figure 2.**
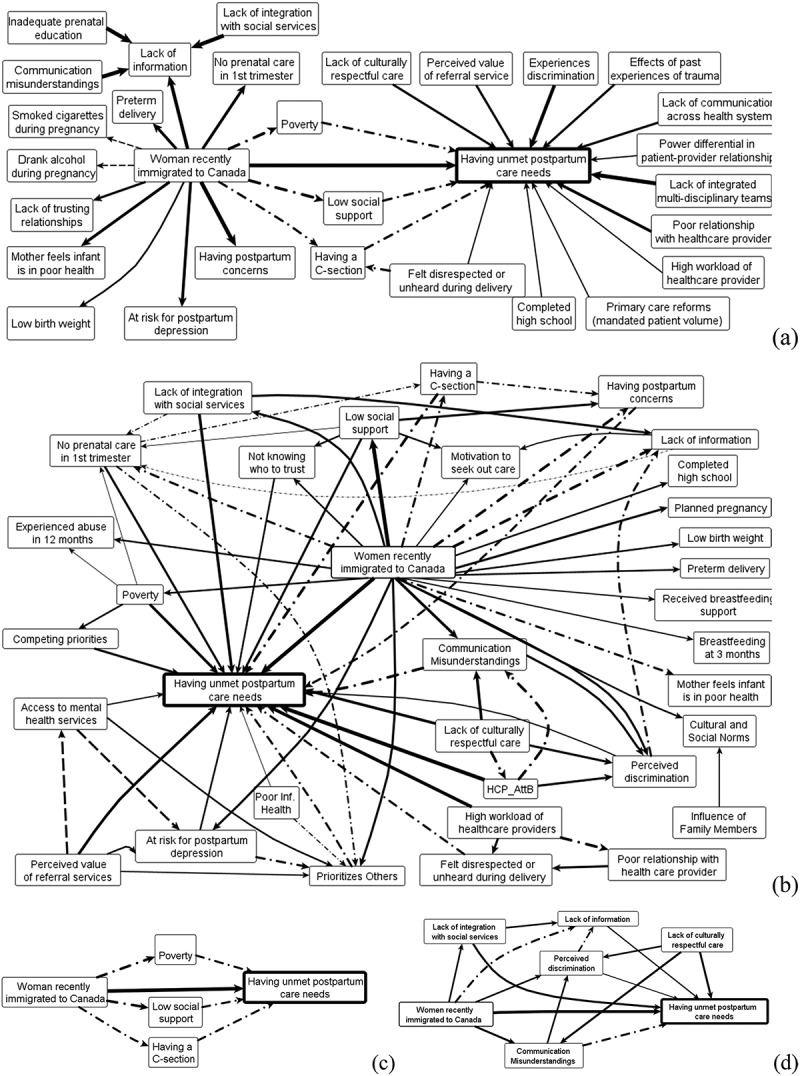
Causes of unmet postpartum care needs among recent immigrant women in Canada according to two groups of stakeholders.

Legend. The transitive closure maps illustrate the perspectives of a family physician (a) and a birth companion (b). Dash-dot-dashed lines correspond to the arrows identified after calculating transitive closure. Dashed lines signify a negative relationship, where an increase in the cause leads to a decrease in the outcome and vice versa. Panels (c) and (d) showcase subsets of causal pathways singled out by family physicians and birth companions, respectively.

Our open-source software integrates transitive closure computation (CIETmap downloadable free at www.ciet.org/fcm). After finalizing our standardized adjacency matrices, we specify probabilistic transitive closure (ProbTC) or fuzzy transitive closure (FuzzyTC). The package can combine several maps and returns a list of the most influential causality ‘walks’. It also shows adjusted weights for positive (TC+) and negative (TC-) relationships, portrayed as two adjacency matrices. The net transitive closure (net TC) results from adding the positive and negative matrices. In Canada, family physicians and birth companions generated separate maps of what each considered causes of unmet postpartum need [22]. Fuzzy transitive closure identified direct and indirect relationships in each map (Figure 2).

A pattern correspondence table *after transitive closure* juxtaposes perceived influences of each factor taking account of all other relationships. Table 2 shows each influence on unmet postpartum care after fuzzy transitive closure, contrasting the knowledge of birth companions, family physicians, and the published literature [22,]. It labels factors mentioned by more than one knowledge source ‘similarities’, and by only one source ‘differences’.

**Table 2.** Pattern correspondence table comparing literature and stakeholder maps on factors influencing unmet postpartum care needs among recent immigrant women.

Factor	Literature	Family Physicians	Birth Companions
**Similarities**			
**Shared by all stakeholders**			
Being an Immigrant	0.2	0.8	1.0
Having a Caesarean Section	0.2	0.5	0.8
Poverty	0.4	0.5	0.5
**Shared by Literature and Family Physicians**			
Less Than High School	0.2	0.3	0.0
**Shared by Family Physicians and Birth Companions**			
Poor relationship with provider	0.0	0.5	0.9
Provider Workload	0.0	0.3	0.6
Lack of Respectful Care	0.0	0.3	0.5
Perceived value of care	0.0	0.4	0.5
Low Social Support	0.0	0.5	0.5
Patient Has No Voice	0.0	0.3	0.3
Perceived Discrimination	0.0	0.6	0.2
Fragmentation between health and social services	0.0	0.5	0.5
**Differences (only one source mentioned it):**			
Lack of multi-disciplinary teams	0.0	0.7	0.0
Communication Misunderstandings	0.0	0.0	0.6
Family Responsibilities	0.0	0.0	0.4
History of Trauma	0.0	0.4	0.0
Experience of Delivery	0.0	0.0	0.4
Risk for Depression	0.0	0.0	0.3
Not Knowing Who to Trust	0.0	0.0	0.3
Lack of Access to Mental Health Services	0.0	0.0	0.3
**Average degree of disagreement on factors influencing unmet postpartum care needs**			
Between Family Physicians and Birth Companions			0.3
Between Family Physicians and the Literature		0.4	
Between Birth Companions and the Literature			0.4

Legend. Reproduced under the authorization of the authors of [22].

The table also shows the level of disagreement about influences on the main outcome (unmet postpartum care needs) across knowledge sources. Disagreement increases with the frequency of different weights and connections, and it decreases with more frequent similar connections. We define the average degree of disagreement ($\overset{ˉ}{d}$) as:

(1) $$\overset{ˉ}{d}=\frac{\sum^{\left|d\right|}}{N},$$

in the equation, *|d|* is the absolute difference of *net transitive closure weights* of the same link in two knowledge sources. Caesarean section had post-transitive closure influences on unmet postpartum care needs of 0.2 in literature maps and 0.8 in maps of childbirth companions (Table 2). The absolute difference is 0.6. Equation 1 adds all the absolute differences between two knowledge sources and divides them by the number of compared relationships (*N*). A higher value of $\overset{ˉ}{d}$ indicates a greater difference between knowledge sources. For comparisons, one can normalise differences dividing $\overset{ˉ}{d}$ by two, the maximum level possible of average disagreement, which results from all relationships having a positive weight of one, on one map, and all the relationships on the other map having a negative weight of one. Relationships with stronger weights and lower disagreement correspond to strong determinants [32]. We can also compare disagreement over entire maps. To do this, we divide the sum of absolute differences across all the reported relationships ($\sum^{\left|d\right|})$ by the number of all possible relationships on the maps. This is equal to the total number of cells in the adjacency matrix or the number of factors on the map raised to two (*k*^*2*^). Higher values show how both the number of relationships and their weights differ across maps.

In stakeholder maps, the authors usually assign weights to reflect the influence they consider each factor has on its corresponding effect. In some cultures, including older Inuit in northern Canada, views of causality do not fit with weighting individual causal components – the *entire map* is a single causal dynamic. A more common reason for not having participant weights is logistical. It takes more (usually unpaid) time of participants. People get tired. In one research setting, participant-assigned weights were not available and researchers generated weights relying on their own appreciation of participant emphasis while building the map [36]. This operator dependence introduces variability and unquantifiable bias.

An alternative operator-independent weighting strategy draws on discourse analysis as proposed by linguist Zellig Harris in the 1950s [37]. This derives structural meaning from the *relative frequency* of each discourse element or morpheme (the units of irreducible meaning composed of several words, a word, or a part of it). Our application of Harris’ approach uses node-arrow-node relationships as the unit of meaning in cognitive maps. Fuzzification is straightforward, counting the frequency of morphemes (relationships in each map) and comparing their relative frequency across several transitive closure maps. We tested this approach in maps of community members in Nigeria [25] and intercultural researchers in Mexico [38]. In both cases, the results of participant and operator-independent weighting had close similarity.

### Moment 3: simplify maps for communication

Some maps contain hundreds of concepts and relationships. Even highlighting the most influential ones, it can still be too much information to guide what to do first or what would be more effective. Stakeholder maps often identify several factors with related implications for action. *Reduction* creates simpler maps. It can include *categorization* to group factors into themes. *Condensation then calculates the net influence of each category* or *aggregation* estimates the average influence of the factors in each category. Finally, we can use *restriction* to display only the strongest relationships, ignoring the weaker ones. In all cases, labelling maps with care will remind users that several relationships and complex interactions remain hidden.

*Categorization* clusters factors into themes, as one might do in conventional thematic analysis [39,40]. Within the categories, we keep original labels assigned to each factor during the mapping sessions. This helps definition of the themes. A common challenge in any thematic analysis is that researchers adopt categories that do not fully reflect participant ways of seeing things. We try to involve map authors in categorization, although this is not always possible. Detailed documentation of the discussion during the mapping session can help make sense of the factors and how best to group them. If map authors are not available, FCM facilitators might contribute their perspectives to categorization. In our FCM protocol, categorization occurs after the initial standardization of factors (see Moment 1). Member checking is a good strategy, engaging similar people when map participants are not available [41]. An important lesson is we cannot interpret category-level relationships in the same way as the factor-level relationships within them. We try to be very transparent in reporting two levels of analysis, clarifying if we are describing factor-level or category-level results.

A study of Hausa community views about causes of short birth intervals in Northern Nigeria generated maps from 52 groups in 10 communities. It was not feasible for all these groups to participate in categorization. The local research team, including facilitators and reporters from the mapping sessions, organized individual factors into categories, referring to notes of the mapping sessions as necessary. They later visited the communities that built the maps for member-checking the results [25].

In Mexico, a group of researchers with experience in Indigenous health and map facilitators together proposed and refined categories [42] to summarise traditional midwives’ maps (Table 1). After this analysis phase, member checking [41] with the authors of the maps confirmed if they agreed with the categorization. Table 1 presents 12 categories summarising 20 unique factors from the maps in Mexico. This table documents assumptions throughout the process, even registering who participated in different decisions.

With categorization completed, we calculate category-level weights. Each category is a subgraph of the map involving all the nodes within it, and we can show this as a single category node [43]. The arrows in subgraph are a ‘self-loop’. These reflect the internal dynamic in the category according to the original map. If category-level relationships have strong weights, reverting to the original map identifies relevant factors with the strongest influence. We can calculate category-level weights [38], the net influence of each category, as the sum of all relationships in the category. Another option is to *aggregate* factor-level relationships to calculate the category-level weights as the average of possible relationships in the category. We can upgrade the averaging to weighted averaging by assigning a relevance factor to each original node, more important nodes having a larger relevance factor. We handle positive and negative weights separately using the TC+ and TC- matrices, so we need not mention the signs. A more formal, detailed description of this operation is Appendix 3.

*Restriction* limits the number of relationships for discussion by showing only the most influential. We can choose to show weights above a certain value or to those linked with nodes that have a greater influence on the main outcome on the map. One can restrict both factor- and category-level maps. In Nigeria, we restricted category maps to the five strongest perceived causes of short birth intervals [25]. We used line styles to show positive and negative influences, with the thickness of arrows proportional to their weights to support participation independent of education level. We use graphic formats for restricted maps, but we also provide adjacency matrices listing all relationships to ensure transparency of results.

### Moment 4: identify priorities for action

In participatory research, we ask FCM participants to weigh each cause–effect relationship in their map by their perception of its influence [1]. Social network analysis (SNA) is hard to interpret before transitive closure since, despite looking like a network, participants build the cognitive map as a series of individual associations. Transitive closure converts the individual weighted associations into a knowledge network. We calculate SNA parameters [4,44,45] only *after* transitive closure.

Several free software options compute standard SNA measures with accepted interpretation (for example, R packages netrankr [46], igraph [47] and FCMapper [48]). We use yEd, a no-cost software for creating graphs that offers the measures we find the most informative [49]:

*Degree centrality* reflects a node’s importance calculated as the number of arrows linked to it. *Indegree centrality* counts incoming arrows and *outdegree centrality* counts outgoing arrows [45]. More prominent outcomes (with more incoming arrows or influences) have a higher indegree centrality, and more important causes (with more outgoing arrows or influences) have a higher outdegree centrality [4].

*Weighted centrality* calculates indegree, outdegree, or overall degree but considering the *absolute value* of the weight for each arrow [50]. The interpretation of this measure is like degree centrality. Even if two nodes have the same degree centrality because they have the same number of incoming or outgoing arrows, the node linked with stronger arrows (higher weights) will have a higher weight centrality.

*Betweenness centrality* reflects how often a node lies on the shortest path between a pair of nodes, in relation to all pairs of nodes on the map. For this measure, higher values suggest that the node is an important modifier of the relationships between other nodes.

**Figure 3.**
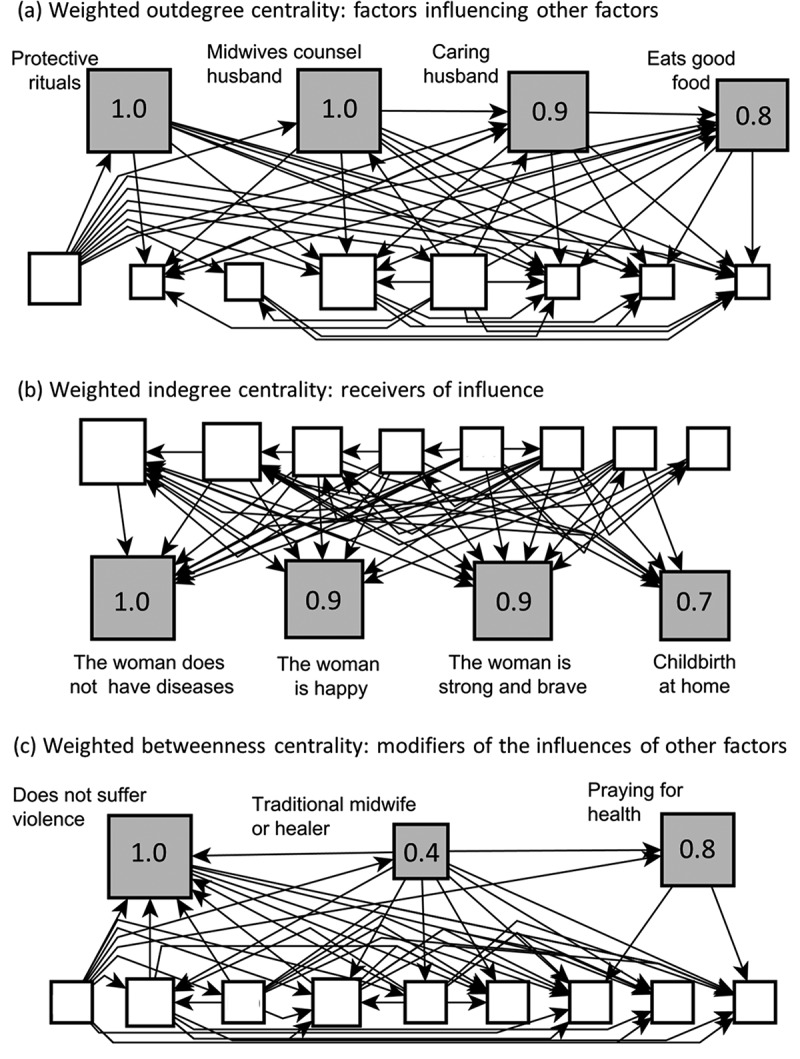
Weighted centrality measures of the map on protective factors for maternal health according to a group of traditional midwives in Guerrero, Mexico.

Legend. Node sizes correspond to the degree of centrality of protective factors in each measurement, with larger boxes denoting higher degrees. Gray boxes are the strongest nodes on the map. The figures inside the grey boxes correspond to the scale for the node’s centrality measure. The other boxes correspond to the rest of the factors on the map, and we left their labels blank to avoid unnecessary details.

Figure 3 shows weighted centrality and betweenness centrality measures for a factor-level map from the *Nacue ñomnda* in Guerrero. yEd calculated these measures for all nodes. The package scaled measures for each node from zero to one, for interpretation like the probability scale. A value of one is the maximum across all the nodes, other values reflecting the proportion of the centrality measure related to that maximum. In the graphic format, the size of the nodes reflects the centrality measures. The biggest node has the highest value, and sizes of other nodes decrease proportionately for lower values.

## Discussion

Four complementary moments in analysis help to make the most of fuzzy cognitive mapping in participatory research. These moments address weighted relationships [51] by combining graph theory [43], fuzzy logic [52] and social network analysis [45]. Drawing on advanced computation, free digital tools increase access for users without the high levels of training in computational sciences. Applying the four analytical moments described here, we also applied FCM for stakeholder input in questionnaire design, co-design of interventions, contextualization of systematic literature reviews and advanced Bayesian statistical analysis [16].

Juxtaposition of different views of causality in a pattern matching table identifies similarities and differences [53]. This requires careful documentation of researcher assumptions or work with map authors to clarify factor labels. Without understating the merits of computer-free analysis, transitive closure adds considerable value where computing aids are available, accounting for interdependence of factors in the maps. This turns the factor map into a knowledge network of direct and indirect connections between factors. Inclusion of the indirect paths between nodes allows social network analysis to recognize mechanisms of influence that might not be immediately evident even to the authors of the maps.

Reduced maps have fewer elements to communicate and, while losing detail, fuel discussion of complex action-oriented ideas [3,50]. Standard qualitative research procedures can apply to thematic analysis of maps. This improves with member-checking. We use different levels of analysis (factor versus category) to see the big picture of causality contained in the maps and then to focus on the details of specific causal mechanisms. We should not confuse inference at the category level (groups of related factors) with factor level influences [43].

We do not know the ideal or the minimum number of maps for analysis. In participatory research, the number of different voices to be heard is the first consideration. In our experience, an optimal solution is continued map building until reaching saturation of new concepts, which usually requires 12 to 15 maps for each stakeholder group. Other researchers have reported similar numbers for focus group discussions and interviews [54,55].

Appendix 2 lists other techniques for FCM analysis, some of which fit the participatory research setting. In AI-assisted computation, maps can also frame simulation models with learning algorithms [11,56–58].

Our practice of comparing the themes reported across knowledge sources and calculating transitive closure to convert the map into a knowledge network makes a good grounding for other approaches. The moments we describe use free computation options. The assumptions and implications of each analytic moment are straightforward for participants, local researchers and practitioners of participatory research. FCM is robust to differences in education, culture and social setting. Combined with accessible analysis, FCM provides space for marginalized subgroups to contribute their views evidence, to make sense of their own experience, and thus to decision-making and action to address the issue at hand.

When interpreting FCM, it is important to recognize that the models developed by participants do not always reflect true causality. Stakeholder maps tell us what those specific stakeholders believe about causality, and that can be very useful to support dialogue and develop intervention strategies in their setting. In participatory research, FCM informs dialogue between stakeholders and their knowledges reflected in different maps. We hope these analytical moments extend the base of local researchers and users capable of engaging in formal dialogue across different perspectives.

## Supplementary Material

Supplemental Material
