# Supplementary material for: Four analysis moments for fuzzy cognitive mapping in participatory research
Source: Glob Health Action. 2024 Dec 2;17(1):2430024. doi: 10.1080/16549716.2024.2430024 (PMC11613336; doi:10.1080/16549716.2024.2430024)

### Appendix 1. Four representations of a fuzzy cognitive map

Indigenous traditional midwives in Guerrero, Mexico, drew a fuzzy cognitive map in a four-hour session with the support of a facilitator and two Indigenous intercultural brokers. The map presents protective factors for maternal health according to participants. We present four formats to depict the same information about a map. The map has 12 concepts (nodes) and 38 relationships (arrows). We present two graphical depictions: a) an original map with labels in Spanish drawn during a group mapping session; b) a digitized diagram drawn in the free software yEd. Two additional tabular formats are c) an adjacency matrix of twelve rows and columns, one per each node in the map, and d) an edge list of the 38 relationships.


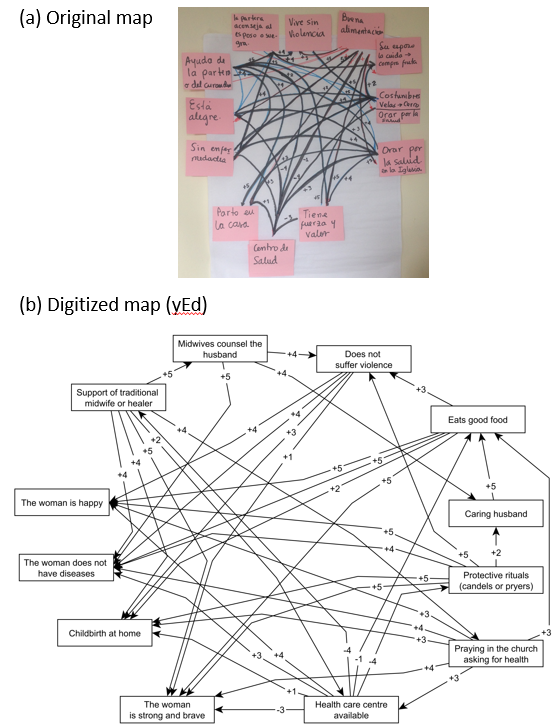


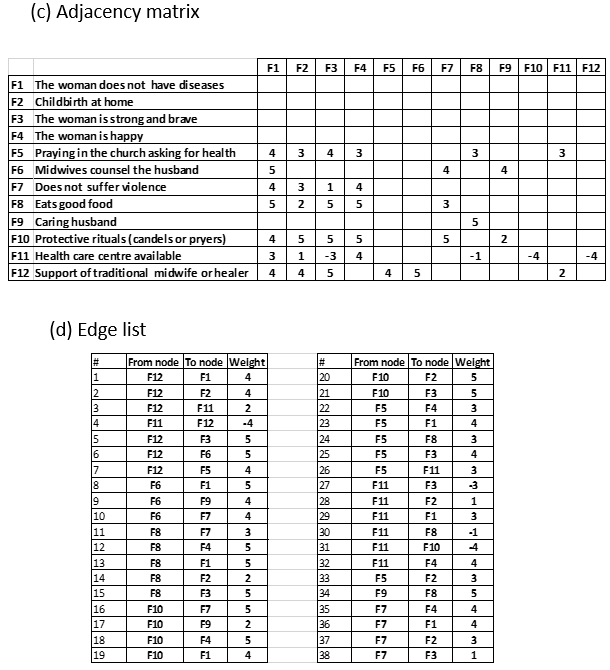

Supplement: Supplemental Material [file ZGHA_A_2430024_SM9986.zip › Appendices/FCM_Appendix1.docx]
