# Supplementary material for: Four analysis moments for fuzzy cognitive mapping in participatory research
Source: Glob Health Action. 2024 Dec 2;17(1):2430024. doi: 10.1080/16549716.2024.2430024 (PMC11613336; doi:10.1080/16549716.2024.2430024)
Supplement: Supplemental Material [file ZGHA_A_2430024_SM9986.zip › Appendices/FCM_Appendix2.docx]

### Appendix 2. Other Approaches to the Analysing of fuzzy cognitive maps

*Simulation with activation rules*

User-specifications (also known as activation rules) are often used to iteratively model scenarios in fuzzy cognitive mapping (FCM) (1,2). This Appendix outlines how activation rules are used to model scenarios and describes select software tools to support FCM analysis. Key resources we have found helpful for additional background include Kosko for the mathematical origin of the procedure (3), Felix et al. for complete review of the evolution of this approach (4), and Peña et al. (5) and Ozesmi & Ozesmi (6) for step-by-step descriptions of completing the mapping with and without stakeholder involvement. Iterative simulations drawing on user-led activation rules offer opportunities for algorithmic learning by calculating new weights based on each new additional simulation (7).Users must specify the extent to which one node affects another (describing their activation state as for example, 0 for no effect and 1 for total effect) for each relationship between nodes within the initial model, at time *t.* Inter-dependence between nodes can also be modelled using “if-then” scenarios, if for example the activation of one node is dependent on the activation state of other surrounding nodes. Activation states are iteratively recalculated using a monotonic function summing the overall effects entering a node until the system stabilizes, meaning values in each subsequent iteration are almost the same as the those in the previous iteration. Alternatively, users can specify that the algorithm complete a maximum number of iterations. Effects on a specific node are calculated as the product of the level of activation of the origin node times the weight of the relationship between nodes (8). See Felix for a helpful summary of the most common activation rules (4)

There are many open-access software packages to support FCM analysis. These include: FCM Wizard (9), FCM Expert (10) or FCM (11) and FCMapper (12) packages in R. Mental Modeler (13) also offers a user-friendly interface to create maps and develop visual representations of iterative simulations in real-time. This has been an especially useful feature to display different scenarios with decision-makers and communities, as well as in teaching scenarios.

*Social network analysis*

Social network also offers several analytical approaches to compare maps (14). For example, indegree centrality measures can help identify different ways in which nodes influence others within a network, such as a transmitter or receiver (6, 15). Density, the proportion of edges in relation to the total possible edges within the network, and centrality, the ratio of transmitter to receiver nodes, are sometimes used to assess the complexity of maps. Maps built together with stakeholders and communities tend to vary based on the quality and length of the mapping session, and therefore we tend not to use these measures to compare maps.

*Comparing maps*

Another approach to comparing maps accounts for weights of relationships between nodes and variability in map structure (16). This approach identifies nodes that are strong, weak and controversial based on a) levels of consensus about their role and influence across compared maps, b) overall importance of the node across compared maps, and c) a measure of heterogeneity in these two measures across compared maps. An overall measure of the level of agreement between maps can be calculated by 1) calculating the difference in net influence each node has on the outcome after transitive closure, and 2) averaging the absolute value of these differences. Our use of this approach was helpful to identify (a) validated connections where compared maps share a non-zero connection of the same direction (e.g., positive or negative), (b) non-validated connections, where a node is only included in one map, and (c) conflicting connections, where multiple maps include the relationship, but are assigned different directions (17). These analytical techniques can be applied to original as well as condensed maps to compare categories.

**References**

1. Aguilar J. A survey about fuzzy cognitive maps papers. Int J Comput Cogn. 2005;3(2):27–33.

2. Papageorgiou EI. Learning Algorithms for Fuzzy Cognitive Maps—A Review Study. IEEE Trans Syst Man, Cybern Part C (Applications Rev. 2012 Mar;42(2):150–63.

3. Kosko B. Hidden patterns in combined and adaptive knowledge networks. Int J Approx Reason [Internet]. 1988 Oct 1 [cited 2019 Apr 9];2(4):377–93. Available from: https://www.sciencedirect.com/science/article/pii/0888613X88901119

4. Felix G, Nápoles G, Falcon R, Froelich W, Vanhoof K, Bello R. A review on methods and software for fuzzy cognitive maps. Artif Intell Rev [Internet]. 2019 Oct 17;52(3):1707–37. Available from: http://link.springer.com/10.1007/s10462-017-9575-1

5. Peña A, Sossa H, Gutiérrez A. Knowledge and Reasoning Supported by Cognitive Maps. In: Mexican International Conference on Artificial Intelligence [Internet]. Springer; 2005. p. 41–50. Available from: http://link.springer.com/10.1007/11579427_5

6. Özesmi U, Özesmi SL. Ecological models based on people’s knowledge: a multi-step fuzzy cognitive mapping approach. Ecol Modell [Internet]. 2004 Aug;176(1–2):43–64. Available from: https://linkinghub.elsevier.com/retrieve/pii/S030438000300543X

7. Poczeta K, Yastrebov A. Analysis of fuzzy cognitive maps with multi-step learning algorithms in valuation of owner-occupied homes. In: IEEE International Conference on Fuzzy Systems, FUZZ-IEEE 2014. 2014. p. 1029–35.

8. Kosko B. Fuzzy knowledge combination. Int J Intell Syst [Internet]. 1986;1(4):293–320. Available from: http://doi.wiley.com/10.1002/int.4550010405

9. Papageorgiou K, Carvalho G, Papageorgiou EI, Bochtis D, Stamoulis G. Decision-Making Process for Photovoltaic Solar Energy Sector Development using Fuzzy Cognitive Map Technique. Energies [Internet]. 2020 Mar 19;13(6):1427. Available from: https://www.mdpi.com/1996-1073/13/6/1427

10. Nápoles G, Espinosa ML, Grau I, Vanhoof K. FCM Expert: Software Tool for Scenario Analysis and Pattern Classification Based on Fuzzy Cognitive Maps. Int J Artif Intell Tools [Internet]. 2018 Nov 14;27(07):1860010. Available from: https://www.worldscientific.com/doi/abs/10.1142/S0218213018600102

11. Papageorgiou EI, Dikopoulou Z. Package ‘fcm’ [Internet]. 2017 [cited 2019 Sep 12]. Available from: https://cran.r-project.org/web/packages/fcm/

12. Turney S, Bachhofer M. Package “FCMapper” [Internet]. 2016 [cited 2018 Jun 18]. (R package). Available from: https://cran.r-project.org/web/packages/FCMapper/

13. Gray S, Gray S, Cox LJ, Henly-Shepard S. Mental Modeler: A Fuzzy-Logic Cognitive Mapping Modeling Tool for Adaptive Environmental Management. In: 2013 46th Hawaii International Conference on System Sciences [Internet]. IEEE; 2013. p. 965–73. Available from: http://ieeexplore.ieee.org/document/6479949/

14. Langfield-Smith K, Wirth A. Measuring Differences between Cognitive Maps. J Oper Res Soc [Internet]. 1992 Dec;43(12):1135. Available from: https://www.jstor.org/stable/2584270?origin=crossref

15. Gray S, Chan A, Clark D, Jordan R. Modeling the integration of stakeholder knowledge in social–ecological decision-making: Benefits and limitations to knowledge diversity. Ecol Modell [Internet]. 2012 Mar;229:88–96. Available from: https://linkinghub.elsevier.com/retrieve/pii/S0304380011004716

16. Giles BG, Findlay CS, Haas G, et al. Integrating conventional science and aboriginal perspectives on diabetes using fuzzy cognitive maps. Soc Sci Med. 2007;64:562–576.

17. Sarmiento I, Paredes-Solís S, Dion A*, et al* Maternal health and Indigenous traditional midwives in southern Mexico: contextualisation of a scoping review

*BMJ Open*2021;**11:**e054542. doi: 10.1136/bmjopen-2021-054542
